# Supplementary material for: Trends in Real-World Clinical Outcomes of Patients with Anaplastic Lymphoma Kinase (ALK) Rearranged Non-Small Cell Lung Cancer (NSCLC) Receiving One or More ALK Tyrosine Kinase Inhibitors (TKIs): A Cohort Study in Ontario, Canada
Source: Curr Oncol. 2024 Dec 27;32(1):13. doi: 10.3390/curroncol32010013 (PMC11764221; doi:10.3390/curroncol32010013)

Supplementary Material: Further details on methods

Supplementary A: Data Sources

ICES is an independent, non-profit research institute funded by an annual grant from the Ontario Ministry of Health and Long-Term Care[30]. As a prescribed entity under Ontario's privacy legislation, ICES is authorized to collect and use health care data from all individuals in Ontario, Canada (which includes ~13,5 million residents representing 40% of the Canadian population) with provincial health insurance without individual consent for the purposes of health system analysis, evaluation, and decision support[30]. Secure access to these data is governed by policies and procedures that are approved by the Information and Privacy Commissioner of Ontario[30].

Database	Description
Ontario Cancer Registry (OCR)	Contains information on all cancer diagnosis in Ontario including patient demographics, health care system usage, treatments and vital signs. Captures cancer diagnoses since 1964 and cancer stage data since 2007
Ontario Drug Program (ODB)	Contains information on prescription medications covered by the province, including patient demographics, drug details (name, dosage, and frequency), prescribing patterns, and pharmacy data.
New Drug Funding Program database (NDFP)	Describes the use of intravenous cancer drugs
Activity Level Reporting System (ALR)	Describes chemotherapy and radiation therapy administered in 41 cancer centers and related general hospitals, capturing approximately 60% of the chemotherapy and 100% of the radiation therapy administered in Ontario
CIHI DAD	Includes detailed information on hospital discharges, including patient demographics, diagnoses (ICD codes), procedures (CCI codes), length of stay, and outcomes, supporting health system planning and research
NACRS	Includes data on outpatient services, including emergency department visits and same-day surgeries, capturing patient demographics, clinical conditions, interventions, and visit outcomes, aiding in the evaluation of ambulatory care services.
OHIP	Includes comprehensive records of insured health services provided to Ontarians, encompassing patient demographics, service dates, billing codes for procedures and diagnoses, and healthcare provider information, supporting health system analysis and research.

Confidential

Registered Persons Database (RPDB)	Describes patients' demographic and vital statuses

Access to dataset

The dataset that comprises the total study cohort as defined by the inclusion and exclusion criteria was retrieved and uploaded into ICES's Data and Analytic Virtual Environment, IDAVE, by the ICES team. Within iDAVE, researchers were provided with access to software to perform analyses and create reports. The index date is defined as the diagnosis date of lung cancer. Variables at individual patient level were presented as days from index date. To further maintain patient confidentiality, demographic data such as age and year of diagnosis were provided in categories.

Supplementary B: Inclusion Definitions

- Lung cancer diagnosis was defined using ICD codes ICD-O-3 codes C340-C343 and C348-C349.

- ALK inhibitor treatment was defined using the drug identification numbers of approved ALK inhibitors in Canada as follows: Alectinib / Alecensaro / DIN 02458136; Ceritinib / Zykadia / DIN 02436779; Crizotinib / Xalkori / DIN 02384256 - 200 mg; 02384264 - 250 mg; Brigatinib /Alunbrig / DIN 02479206 - 30mg; 02479214 - 90mg; 02479222 - 180mg; 02479230 - 90mg/180mg; and Lorlatinib / Lorbrena / DIN 02485966; 02485974.

- Chemotherapy regimens from ALR included the following CCO chemotherapy treatments and treatment combinations:*CISPETOP(3D), *CISPETOP(RT), *CISPGEMC, *CISPGEMC, *CISPEME, *CISPEME(RT), *CISVINO, *CISVINO(RT), *CISVINO(W), *CISPVNBL(RT), CRBPETOP, *CRBPETOP(PO), *CRBPETOP(RT), *CRBPRACL, *CRBPRACL(RT),*CRBPRACL(W), *CRBPPEME, *CRBPPEME(RT), *CRBPVINO, *CRBPVNBL, *DEXALENA, *DEXAPOMA, *DOCE, *GEMC, *GEM-CARBO, *PEME, *PEME(MNT), *PEMETREXED, *VINOCISP and *CRBPPEME

Confidential

Supplementary C: Complete listing of rules to determine line of treatment

Determining treatments, treatment duration, total lines of treatments

Treatment regimens at individual patient level were retrieved from the ODB and the ALR databases. A lung cancer treatment regimen may include chemotherapy, immunotherapy, ALK TKIs, other targeted therapy or clinical trial therapy. The 'DIN number' data variable was used to identify ALK TKIs from the ODB and ALR databases. The "CCO_regimen' data variable was used to identify chemotherapy from the ALR database. Chemotherapy included different types of chemotherapy treatment(s) and treatment combinations (see Supplementary Section B).

Treatment duration was derived based on the first and last administration dates of a treatment regimen. The data variable used to determine administration dates is 'days_to_serve_date' from ODB or 'days_to_visit_date' from ALR. For a patient to be considered to have received a lung cancer treatment regimen, more than one 'days_to_serve/visit' date for a treatment must be available. This is to ensure that patients indeed received the drug. The smallest value of 'days_to_serve/visit date', the largest value of 'days_to_serve/visit date' and the number_of_days_supplied were reviewed for each treatment regimen. Treatment start was defined as the smallest value of 'days_to_serve/visit date'. Treatment end was defined as the largest value of 'days_to_serve/visit date' plus the number of days supplied. One line of treatment was assumed for patients that received chemotherapy doublet treatment followed by chemotherapy

monotherapy treatment. Treatment duration was defined as the (treatment end + days supplied) subtracted from the (treatment start).

The total lines of treatment settings were established at individual patient level. This is defined as the total number of treatment regimens that a patient received during the study. In addition to ODB, ALR contained information on ALK TKIs that were not available in ODB. For the purpose of calculating the total lines of treatment settings, data on ALK TKIs from ALR were also considered and presented as part of the total lines of treatment settings count.

Listing of rules to determine line of treatment

- Requires the use of 2 reports (ODB and ALR) - data sources

ODB:

- Pull smallest value of ‘days_to_serv_date’ for
  - DINs 02458136, 02436779, 02384256 and/or 02384264
- DINs 02479214, 02479222, 02479230 (Brigatinib) not found in ODB
- DINs 02485966 and 02485974 (Lorlatinib) not found in ODB

ALR:

- Pull smallest value of ‘days_to_vist_date’ for CCO_regimens:
  - *CRIZ, *ALEC, *CERI, *BRIG, and *LORL
- Pull smallest value of ‘days_to_visit_date’ for CCO_regimen:
  - *CAV, *CISPETOP(3D), *CISPETOP(RT), *CISPGEMC, *CISPGEMC, *CISPEME, *CISPEME(RT), *CISVINO, *CISVINO(RT), *CISVINO(W), *CISPVNBL(RT), CRBPETOP, *CRBPETOP(PO), *CRBPETOP(RT), *CRBPRACL, *CRBPRACL(RT),*CRBPRACL(W), *CRBPPEME, *CRBPPEME(RT), *CRBPPME+PEMB, *CRBPVINO, *CRBPVNBL, *DEXALENA, *DEXAPOMA, *DOCE, *GEMC, *GEM-CARBO, *PEME, *PEME(MNT), *PEMETREXED, *VINOCISP and *CRBPPEME

Confidentia

For each patient (for each unique ICES encryption number), treatment start is:

- DIN or CCO_regimen with the smallest value of ‘days_to_visit_date’ or ‘days_to_serv_date’

For each patient (for each unique ICES encryption number), treatment stop is:
ODB:

- Add 30 days to largest value of ‘days_to_serv_date’ for
  - DINs 02458136, 02436779, 02384256 and/or 02384264

ALR:

- Add 30 days to largest value of ‘days_to_vist_date’ for CCO_regimens:
  - *CRIZ, *ALEC, *CERI, *BRIG, and *LORL
- Add 21 days to largest value of ‘days_to_visit_date’ for CCO_regimen:
  - *CAV, *CISPETOP(3D), *CISPETOP(RT), *CISPGEMC, *CISPGEMC, *CISPEME, *CISPEME(RT), *CISVINO, *CISVINO(RT), *CISVINO(W), *CISPVNBL(RT), CRBPETOP, *CRBPETOP(PO), *CRBPETOP(RT), *CRBPRACL, *CRBPRACL(RT),*CRBPRACL(W), *CRBPPEME, *CRBPPEME(RT), *CRBPPME+PEMB, *CRBPVINO, *CRBPVNBL, *DEXALENA, *DEXAPOMA, *DOCE, *GEMC, *GEM-CARBO, *PEME, *PEME(MNT), *PEMETREXED, *VINOCISP, CRBPPEME, and/or CARBPPEME+PEMB

For each patient (for each unique ICES encryption number), List the line of each treatment setting based on below:

- DIN or CCO_regimen with the smallest value of ‘days_to_serv_date’ or ‘days_to_visit_date’ = 1st line of systemic treatment
- DIN or CCO_regimen with the 2nd smallest value of ‘days_to_serv_date’ or ‘days_to_visit_date’ = 2nd line of systemic treatment
- DIN or CCO_regimen with the 3rd smallest value of ‘days_to_serv_date’ or ‘days_to_visit_date’ = 3rd line of systemic treatment
- DIN or CCO_regimen with the largest value ‘days_to_serv_date’ or ‘days_to_visit_date’ = last line of systemic treatment

Additional Rules

- Additional rules implemented post data mining:
    - Patients must have received 2 scripts of treatment to count as a line of treatment. Otherwise, exclude treatment. This is to ensure that patients indeed received the drug.
    - Patients that receive doublet chemotherapy followed by chemo monotherapy, count as 1 line of treatment.
    - If the last administration date of a first instance of chemotherapy or immunotherapy treatment is >/= 365 days from next treatment, do not include the patient in the First-line Study Cohort as it is not clear whether the patient received 1st instance in the adjuvant or advanced setting.
    - In ODB, ALK inhibitors were not always supplied in monthly increments. Many times, ALK inhibitors were supplied for 60, 90 or 120 days. As a results:
        - Use the last ‘days supplied’ if </= 30 days, round to 30 days (default).
        - If the last claim supplied for > 30 days, then examine the previous trend of ‘days supplied’. If the last claim is > than all previous ‘days supplied’, use previously supplied days. If the trend of ‘days supplied’ is similar to the last claim, then use the last claim of ‘days supplied' (which may be 60, 90 or 120 days).
        - Intent is to estimate duration of treatment appropriately and therefore, had to customize originally planned duration of treatment calculation based on data in reports.
    - Various additional ALK inhibitors were found in ALR but not in ODB, treatments were included in lines of treatment count.
    - Immunotherapy, targeted therapy and clinical trials were also found in ALR database, those treatments were also counted in lines of treatment count.
    - If 2 different treatments overlap in serving date between ODB and ALR, use ODB.
    - If serv dates in ALR were earlier than ODB, the earlier serve dates from ALR were included.

Confidentia

Supplementary D: Plot of the Kaplan-Meier survival curves for different groups

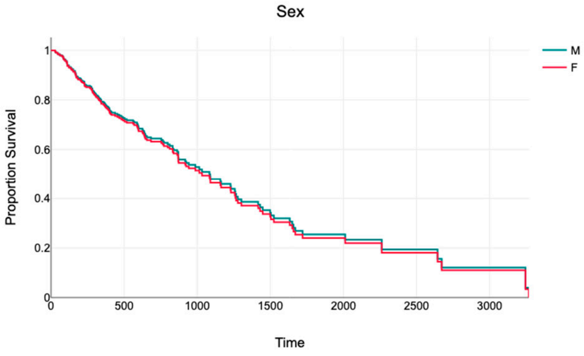

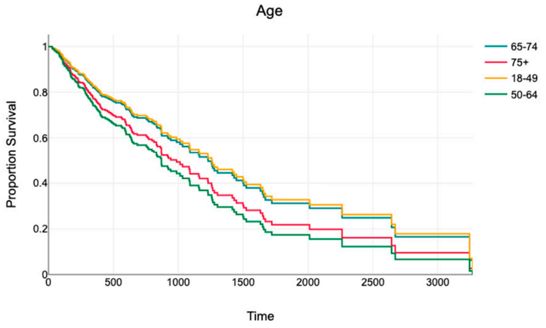

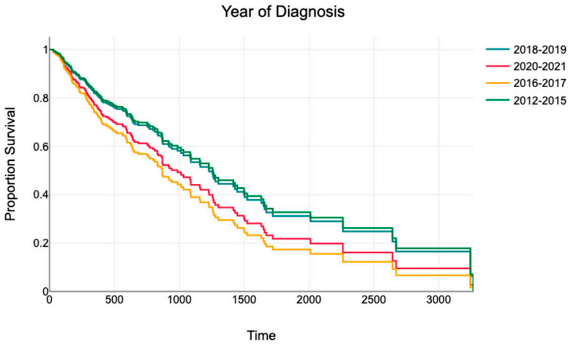

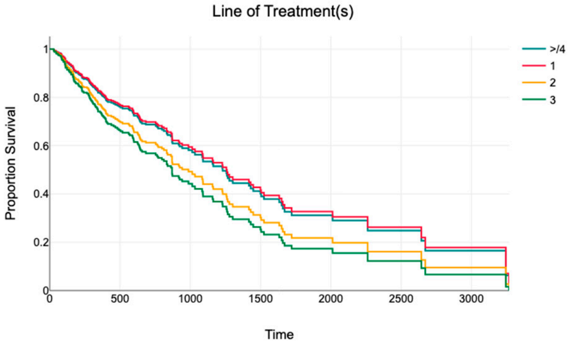

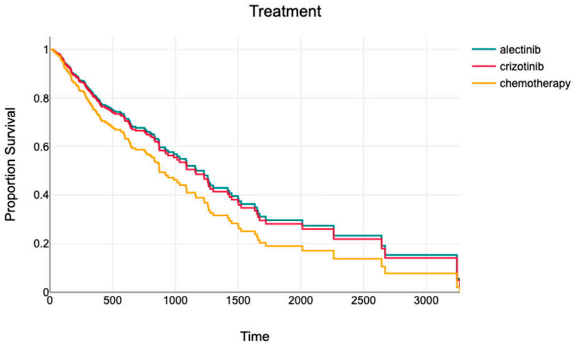

Supplement: Supplementary file 1 [file curroncol-32-00013-s001.zip › curroncol-3364151-supplementary.pdf]
